# *PRM1* Gene Expression and Its Protein Abundance in Frozen-Thawed Spermatozoa as Potential Fertility Markers in Breeding Bulls

**DOI:** 10.3390/vetsci9030111

**Published:** 2022-03-03

**Authors:** Berlin Pandapotan Pardede, Muhammad Agil, Ni Wayan Kurniani Karja, Cece Sumantri, Iman Supriatna, Bambang Purwantara

**Affiliations:** 1 Department of Veterinary Clinic, Reproduction, and Pathology, Faculty of Veterinary Medicine, IPB University, Bogor 16680, Indonesia; rhinogil@apps.ipb.ac.id (M.A.); karja_nwk@apps.ipb.ac.id (N.W.K.K.); imansu@apps.ipb.ac.id (I.S.); 2 Department of Animal Production and Technology, Faculty of Animal Science, IPB University, Bogor 16680, Indonesia; ceces@apps.ipb.ac.id

**Keywords:** fertility marker, frozen-thawed spermatozoa, gene expression, breeding bulls, protamine-1, protein abundance

## Abstract

Functional genes and proteins in sperm play an essential role in bulls’ reproductive processes. They are more accurate in determining bull fertility than conventional semen quality tests. Protamine-1 (*PRM1*) is a gene or protein crucial for packaging and protecting sperm DNA until fertilization affects normal sperm function. This study analyzes the genes and proteins potential from *PRM1* as fertility markers for different breeds of bulls utilized in the artificial insemination programs, expected to be an accurate tool in interpreting bull fertility in Indonesia. This study used Limousin, Holstein, and Ongole Grade bulls divided into two groups based on fertility, high-fertility (HF) and low fertility (LF). The semen quality assessment included progressive motility (computer-assisted semen analysis), viability (eosin-nigrosine), and plasma membrane integrity (HOS test). Sperm DNA fragmentation (SDF) was assessed using the acridine orange staining and the Halomax test. Sperm *PRM* deficiency was evaluated with the chromomycin A3 method. Moreover, *PRM1* gene expression was measured using qRT-PCR, and the *PRM1* protein abundance was measured with the enzyme immunoassay method. Semen quality values, relative expression of *PRM1* gene, and quantity of *PRM1* protein were significantly higher (*p* < 0.05) in HF bulls than in LF bulls. The SDF and *PRM* deficiency values in LF bulls were significantly higher (*p* < 0.05) than HF bulls. Additionally, *PRM1* at the gene and protein levels correlated significantly (*p* < 0.01) with fertility. Therefore, *PRM1* is a potential candidate for fertility markers in bulls in Indonesia.

## 1. Introduction

The artificial insemination (AI) program is a proven and successful reproductive technology in increasing population and livestock production [1]. The AI program for cattle uses frozen semen from different breeds of bulls produced at the AI center with specific standards [2]. Generally, sperm motility above 40% with spermatozoa concentration not less than 25 million per insemination dose is used for quality control of frozen semen in AI programs [3]. This quality standard should predict bull fertility and optimize AI success via a high percentage of pregnancy and calf births. Unfortunately, facts reveal that the success of AI is still comparatively low. Rosyada et al. [4] reported that the conception rate of several Holstein bulls was still classified as low fertility (LF), namely, 33.78% from >1000 AI data and 21.15% from <1000 AI data, even though the quality of frozen semen met the standards, including spermatozoa motility above 40%. Pardede et al. [5] also reported that the %CR of bulls in controlled breeding program centers was sub-optimal, at 63% from <1000 AI data. Additionally, Diskin [6] reported that the %CR is categorized as successful in the AI program when it reaches more than 70% of all first inseminated cows.

Over the years, a paradigm has arisen about classical semen quality assessment, including motility, usually less valuable and reliable in predicting male fertility, particularly in bovine species [7], confirmed by Özbek et al. [8]. They revealed that although standard semen assessment visually identifies poor quality sperm, it was insufficient to detect potential subfertile bull markers. Staub and Johnson [9] showed the potential of molecular, cellular, and physiological disorders inhibiting and affecting sperm quality and production, consequently causing infertility for 61 d in the spermatogenesis process occurring in bulls. Specific genes and proteins in sperm, including sperm plasma, have been shown to possess a crucial function in influencing sperm fertility in a bull [10]. These fertility genes or proteins in sperm can be potential molecular markers in determining the reproductive status of bull [11], considered more effective in deciding bull fertility [7,10]. Notably, the protamine (*PRM*) is one of the many genes or proteins reported as potential molecular markers for predicting the fertility of these bulls [10,12,13].

*PRM* is the essential molecule in the arginine-rich nuclear protein involved in sperm DNA packing [14]. Sperm *PRM* sequentially replaces histone somatic cells through a complex process during spermiogenesis [15]. At the spermatid round, histone and non-histone proteins are substituted by transitional proteins [16], which are withdrawn from the compacted chromatin of elongated spermatids and replaced by *PRM* until sperm development [15,16,17]. The alternation process produces highly dense chromatin, which is a basis of germ cell maturation and unique to haploid cells [17,18,19,20,21]. In bulls [20], the *PRM1* gene is essentially critical in normal sperm functioning. However, PRM2 and PRM3 genes have been expressed in bovine testes [22]. *PRM* plays a fundamental role in protecting sperm DNA from damage such as nucleases and other factors [23]. Additionally, switching nuclear proteins to the *PRM* process silences transcription in the spermatozoa and possibly imprint the paternal genome before fertilization [24].

The *PRM* gene or protein is a potential application for determining male fertility [12,13]. Previous studies reported different negative impacts due to a low gene or protein expression of *PRM* on male fertility, causing the decline in semen quality [25,26,27,28,29]. Consistently, clinical studies in humans showed the association of *PRM* with male infertility cases [30]. Dogan et al. [13] reported the protein of *PRM1* reduction in LF bulls, also characterized by an increase in DNA fragmentation. The *PRM1* gene has been highly expressed in high-fertility (HF) Holstein bulls [12]. It reportedly plays an essential role in determining the semen quality of Frieswal crossbred bulls (HF X Sahiwal) [27]. Our previous study also showed *PRM1* as the protein with the most potential determinant of semen quality of various bulls in Indonesia compared to PRM2 and PRM3 variants [20]. However, studies that prove the potential of *PRM1* as a biomarker of bull fertility used for AI in Indonesia have not been reported. This study analyzes the possibility of genes or proteins from *PRM1* as a marker for determining fertility in various bull breeds used for the AI program through the mRNA and protein approach, which should be an accurate tool for interpreting bull fertility.

## 2. Materials and Methods

### 2.1. Experimental Animals

The Indonesian National AI center produced the frozen semen used in this study. The frozen semen sample used in this study is a commercial product from the AI center, where every procedure, from the process of collecting fresh semen to producing it into frozen semen, including the management of bulls’ maintenance, follows the operational standards of the AI center, which is supervised by a veterinarian and has complied with every principle of animal welfare. The semen was obtained from bulls at their sexual maturity and productive phase, at 4–5 years of age. This study used 18 bulls, including six Limousin, Holstein, and Ongole Grade bulls. Furthermore, each breed was grouped into two based on the fertility score, HF (n = 3) and LF (n = 3). The fertility score was based on each bull’s first service %CR; %CR > 70% are categorized as HF bulls, and %CR < 70% is classified as LF bulls [6]. The %CR data was obtained from AI data and pregnancy tests on cows for the last two years at the National Animal Health Information System (iSIKHNAS), Indonesia.

The iSIKHNAS is an animal health information system in all regions in Indonesia, including data on AI programs accompanied by pregnancy data. The results of the analysis carried out on iSIKHNAS data, the number of cows/heifers that were inseminated in each breed of bulls in the study were 143,010 (Limousin), 81,741 (Holstein), and 98,571 (Ongole Grade) cows/heifers. Meanwhile, the number of AI services for each breed of the bull in the study were 197,588 (Limousin), 106,034 (Holstein), and 136,686 (Ongole Grade) services. The percentage of pregnant cows/heifers from the first insemination service of the total AI services (%CR) was analyzed according to Pardede et al. [5].

### 2.2. Frozen Semen Quality

A total of 90 frozen semen straws (five straws/bull) from six Limousin bulls, six Holstein bulls, and six Ongole Grade bulls were used in this stage. First, the frozen semen was thawed in a water bath at 37 °C for 30 s, then put into a microtube for further observation. Next, the sperm’s progressive motility (PM) parameters were analyzed using computer-assisted semen analysis. After that, 10 µL of thawed sperm were dropped onto a slide and covered with a coverslip. Finally, four fields were evaluated with a range of 50–250 sperm cells automatically counted using Sperm Vision (Minitube, Tiefenbach, Germany) [20].

Additionally, sperm viability was evaluated using eosin-nigrosine (0.825 g eosin Y, 5 g nigrosine, 0.375 g Na Citrate, and 100 mL aquadest) staining. First, the sperm viability was evaluated by making a smear on slides from a mixture of 10 µL semen and 10 µL eosin-nigrosine (1:1). Then, the slides were dried using a heating table, observed, and counted under a light microscope with 40× objective magnification. Two hundred sperm cells per slide were evaluated, with live sperm unstained and dead sperm with damaged membranes stained (reddish) [31]. Next, the integrity of the plasma membrane of sperm was evaluated using the HOS (hypoosmotic swelling) test method. Furthermore, 20 µL of semen was mixed into 300 µL of HOS solution (0.735 g Na citrate, 1351 g, and 100 mL aquadest) and incubated in a water bath at 37 °C for 30 min. Then, 10 µL of the mixture was dropped on a slide, covered with a coverslip, and observed under a light microscope with 40× objective magnification. Finally, 200 sperm cells per slide were counted, with a coiled tail indicating sperm of intact plasma membrane integrity [5,32].

### 2.3. Sperm DNA Fragmentation

Sperm DNA fragmentation (SDF) was assessed using acridine orange (AO) staining and the Halomax test. A total of 90 frozen semen straws (five straws/bull) from six Limousin bulls, six Holstein bulls, and six Ongole Grade bulls were used for AO staining. First, a smear was made 5–10 µL of semen on a slide, then fixed in Carnoy’s solution (methanol: glacial acetic acid (3:1)) for 2 h. Next, the slides were stained in a new AO solution (10 mL of 1% acridine orange, 40 mL of 0.1 M citric acid, 2.5 mL Na_2_HPO_4_7H_2_O) for 5 min in a dark room. Next, a coverslip was placed on the slide after washing with distilled water. Finally, 500 sperm cells per slide were observed and counted under a fluorescent microscope using a 490 nm excitation filter and a 530 nm emission filter. The green fluorescence denoted the sperm with mature chromatin and intact DNA integrity, whereas sperm with fragmented DNA integrity was characterized by a reddish-orange fluorescence [33].

Moreover, according to the manufacturer’s protocols, SDF was assessed using the Halomax test (*Bos taurus,* Halotech DNA SL, Madrid, Spain). A total of 90 frozen semen straws (five straws/bull) from six Limousin bulls, six Holstein bulls, and six Ongole Grade bulls were used for this test. First, 25 µL (15–20 × 10^6^ sperm cells/mL) was mixed with 50 µL of liquid agarose in a microtube at 37 °C. A total of 1.5–2 µL of cell suspension was dropped into marked wells on a pre-cooled slide (4 °C), and a coverslip was placed. The slide was incubated in the refrigerator at 4 °C for 5 min. The coverslip was carefully removed from the slide at room temperature (22 °C), and a lysing solution was dripped on the slide horizontally for 5 min and later dried. Next, the slides were washed with distilled water for 5 min and re-dried. Finally, the slides were dehydrated with 70% and 90% ethanol solutions, respectively, for 2 min and dried. The staining process was done by dripping 5 µL of 0.01 mm propidium iodide dye for 5 min in a dark room. Five hundred sperm cells per slide were observed under a fluorescent microscope using a 510–560 nm excitation filter and a 590 nm emission filter. Sperm cells with the fragmented DNA were then denoted by large and dispersed spotty halos, whereas tiny, dense halos represented sperm as the unfragmented DNA [34].

### 2.4. Sperm PRM Deficiency Assay

Sperm *PRM* deficiency was evaluated using the Chromomycin A_3_ (CMA_3_) staining method. A total of 90 frozen semen straws (five straws/bull) from six Limousin bulls, six Holstein bulls, and six Ongole Grade bulls were used in this stage. Sperm *PRM* deficiency was assessed by smear preparations of 5–10 µL of semen on a slide, then fixed in Carnoy’s solution (methanol: glacial acetic acid (3:1)) for five min at 4 °C. Then, the slides were stained in CMA_3_ dye solution (0.25 mg/mL CMA_3_ in McIlvane’s buffer (pH 7.0) supplemented with 10 mM MgCl_2_) for 20 min. Next, the slide was rinsed in McIlvane’s buffer (pH 7.0) and air-dried. Finally, 500 sperm cells per slide were observed under a fluorescent microscope using a 470–490 nm excitation filter and a 510 nm emission filter. Sperm with deficient *PRM* (CMA3+ positive) was indicated by bright green fluorescence on the head, whereas dull green indicated sperm with intact *PRM* (CMA3−negative) [35].

### 2.5. PRM1 Gene Expression Analysis

A total of 144 frozen semen straws (eight straws/bull) from six Limousin bulls, six Holstein bulls, and six Ongole Grade bulls were used in this stage. Next, each frozen semen was thawed in a water bath for 30 s at 37 °C, washed three times with phosphate-buffered saline (PBS), and pelleted (about 25 × 10^6^ sperm cells/mL) by centrifugation at 16,000× *g* for 15 min. After that, the total RNA was extracted using the TRI reagent, as recommended by the manufacturer (Zymo Research, Irvine, CA, USA). The NanoDropTM One/OneC Microvolume UV-Vis Spectrophotometer (Thermo Scientific, Marsiling Industrial Estate Rd 3, Singapore) determined the total RNA amount and purity (Thermo Scientific, Marsiling Industrial Estate Rd 3, Singapore). According to the manufacturer’s protocols, the SensiFASTTM cDNA Synthesis Kit (Bioline Ltd., Memphis, TN, USA) was also used for cDNA synthesis. A total of 20 µL of cDNA obtained from this reaction was ready for RT-PCR. Next, the NanoDrop One/OneC Microvolume UV-Vis Spectrophotometer (Thermo Scientific, Marsiling Industrial Estate Rd 3, Singapore) was used to assess the total cDNA amount and purity. The quantitative real-time PCR (qPCR) was also used to determine the transcripts’ quantity, and the reactions were performed using the SsoFastTM EvaGreen Supermix (Bio-Rad Lab, Hercules, CA, USA). The genes identified in this study were *PRM1* (BC108207; forward: 5′-AGATACCGATGCTCCTCACC-3′ and reverse: 5′-GCAGCACACTCTCCTCCTG-3′) [22] with PPIA (XM_001252921.1; forward: 5′- ATGCTGGCCCCAACACAA-3′ and reverse: 5′-CCCTCTTTCACCTTGCCAAA-3′) as housekeeping gene [27]. The 2^−^^ΔΔCT^ [36] was used to assess the relative expression levels of the *PRM1* gene, which were normalized to the housekeeping gene PPIA expression.

### 2.6. PRM1 Protein Assay

A total of 108 frozen semen straws (six straws/bull) from six Limousin bulls, six Holstein bulls, and six Ongole Grade bulls were used. Each frozen sperm was thawed in a 37 °C water bath for 30 s, washed twice with PBS, and pelleted (about 25 × 10^6^ sperm cells/mL) by centrifugation at 700× *g* for 15 min. The amount of *PRM1* protein was then measured in sperm pellets using the enzyme immunoassay (EIA) technique, according to the manufacturer’s protocols (Bovine *PRM1*, MyBioSource, Inc., San Diego, CA, USA) [20]. The prepared sperm pellets were briefly added to the appropriate wells.

Furthermore, the reaction wells were closed and hatched in an incubator (37 °C) for 90 min. After that, the EIA plate was washed twice, then 100 µL of antibody solution was added and covered with adhesive tape on each well. Next, the EIA plate was re-hatched in the incubator (37 °C) for 60 min and then washed thrice. A total enzyme solution, 100 µL, was poured into each well, closed, and re-hatched in the incubator (37 °C) for 30 min. Washing was repeated five times on the EIA plate; then, color reagent solution was added and hatched again in the incubator (37 °C) in the darkroom for 30 min. Finally, the color C reagent mixture mixed thoroughly in each well. The analysis was conducted using an EIA reader with a wavelength of 450 nm in 10 min.

### 2.7. Statistical Analysis

The fertility score (%CR) data for each bull breed were analyzed using Microsoft Excel (Microsoft Office Pro Plus 2019 Microsoft). The differences in the mean number of breeding, fertility score (%CR), semen quality parameters, SDF, *PRM* deficiency, the abundance of *PRM1* protein, and relative expression of *PRM1* mRNA in the FH and LF bulls were analyzed using the *t*-test method. The Pearson correlation analysis examined the overall association between the parameters evaluated and the bulls’ percentage CR. A scatter plot matrix graph presented the relationship between %CR, *PRM1* protein, and mRNA. The *t*-test method, Pearson correlation analysis, and scatter plot matrix were obtained using the Statistical Package for the Social Sciences (v.25.0, IBM, Armonk, NY, USA). The data were presented in the form of mean ± standard error.

## 3. Results

The results indicated that the %CR obtained from the AI data from each bull breed was significantly different (*p* < 0.05) between HF and LF bulls (Table 1). The semen quality, such as PM, viability, and plasma membrane integrity, showed that the semen quality in HF bulls was greater (*p* < 0.05) than in LF bulls (Table 1).

**Figure 1:**
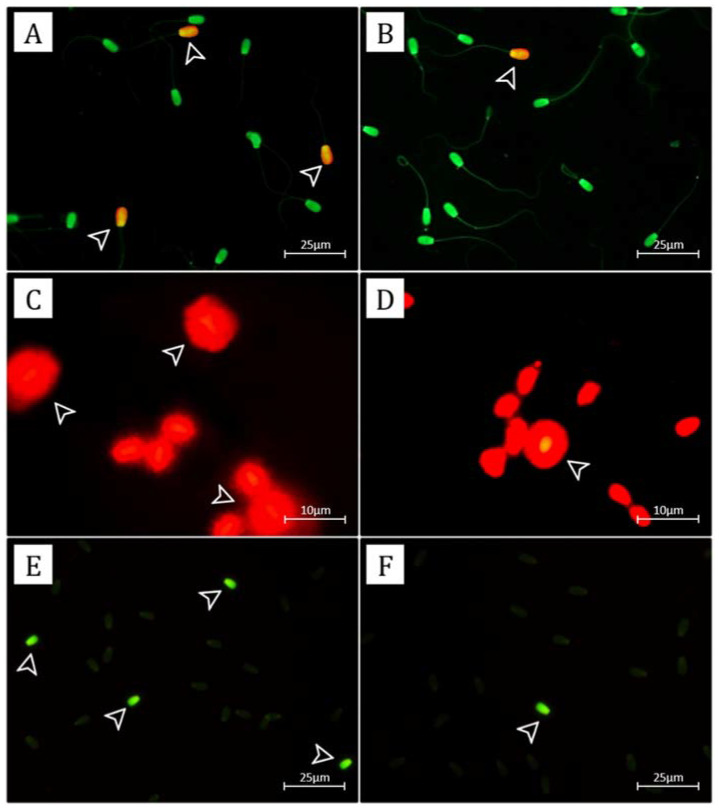
The photomicrograph of sperm in AO staining of HF and LF bulls (**A**,**B**); Sperm with normal DNA integrity will be stained with green fluorescence; SDF will be stained with yellow-orange fluorescence (arrow). The photomicrograph of Halomax test in HF and LF bulls (**C**,**D**); Sperm with normal DNA integrity showed a slight halo, and SDF showed a large halo (arrow). The photomicrograph of *PRM* deficiency by the Chromomycin A3 (CMA_3_) assay (**E**,**F**); bright green fluorescence-stained sperm (CMA3+) indicated *PRM* deficiency (arrow), whereas dull green stained sperm (CMA3−) indicated normal *PRM*-stained sperm.

**Figure 2:**
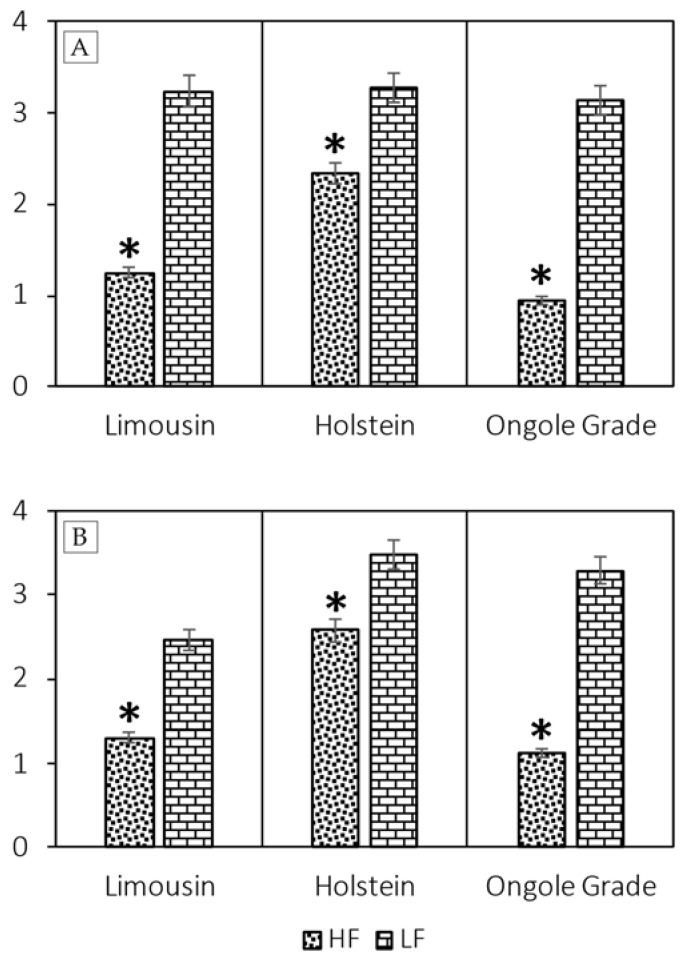
The value of SDF using AO staining (**A**) and Halomax test (**B**) on Limousin, Holstein, and Ongole Grade bulls with different fertility (HF vs. LF). * Significant difference when compared to LF (*p* < 0.05).

The AO staining and the Halomax test was used to evaluated SDF (Figure 1). The assessment of SDF result using AO staining showed that the sperm semen in LF bulls had more SDF than HF bulls (*p* < 0.05) in Limousin (2.38% ± 0.77% vs. 1.25% ± 0.32%), Holstein (3.27% ± 0.63% vs. 2.34% ± 0.34%), and Ongole Grade (3.13% ± 0.79% vs. 0.94% ± 0.36%) bulls (Figure 2A). However, the results of the analysis of SDF using the Halomax test showed that SDF in LF bulls was higher than HF bulls (*p* < 0.05) in Limousin (2.47% ± 1.11% vs. 1.31% ± 0.42%), Holstein (3.49% ± 0.71% vs. 2.58% ± 0.40%), and Ongole Grade (3.29% ± 0.99% vs. 1.13% ± 0.50%) bulls (Figure 2B).

**Figure 3:**
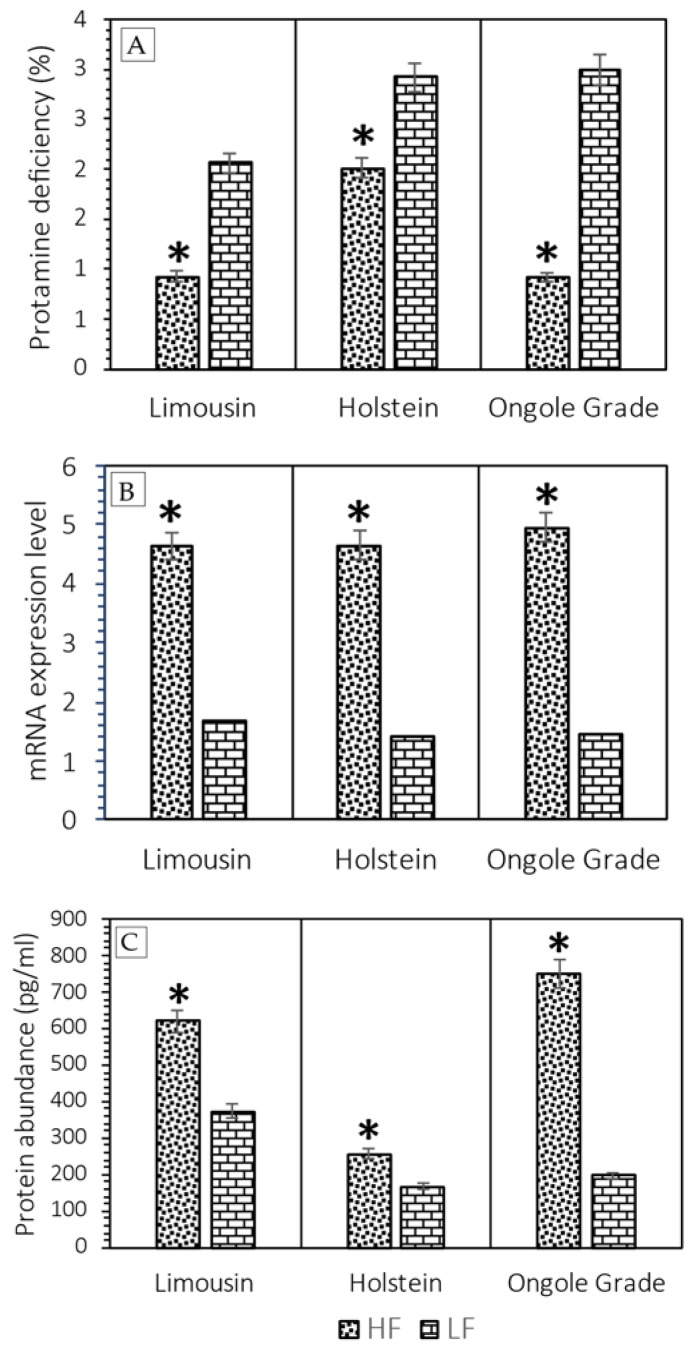
The *PRM* deficiency (CMA3) percentage (**A**), relative expression of *PRM1* gene (**B**), and the abundance of *PRM1* protein (**C**) in Limousin, Holstein, and Ongole Grade bulls with different fertility (HF vs. LF). * Significant difference when compared to LF (*p* < 0.05).

Sperm with *PRM* deficiency conditions were stained bright green fluorescence (CMA3+), and sperm with normal *PRM* were stained dull green (CMA3−) (Figure 1). The assessment of the content of sperm with *PRM* deficiency showed that sperm in LF bulls were significantly more deficient in *PRM* than HF bulls (*p* < 0.05). The mean content of sperm with *PRM* deficiency in LF vs. HF bulls were Limousin (2.06% ± 0.68% vs. 0.93% ± 0.33%), Holstein (2.92% ± 0.50% vs. 2.01% ± 0.37%), and Ongole Grade (3.00% ± 0.74% vs. 0.92% ± 0.42%) (Figure 3A).

The relative gene expression and protein abundance of *PRM1* in each breed in HF bulls were greater (*p* < 0.05) than in LF bulls (Figure 3). The mean relative expression values of the *PRM1* gene in HF vs. LF bulls were Limousin (4.64 ± 0.57 vs. 1.68 ± 1.23), Holstein (4.65 ± 0.61 vs. 1.42 ± 1.04), and Ongole Grade (4.95 ± 0.75 vs. 1.46 ± 1.07) (Figure 3B). Protein abundance of *PRM1* in HF vs. LF bulls was Limousin (621.44 ± 219.37 pg/mL vs. 374.00 ± 257.71 pg/mL), Holstein (257.11 ± 54.28 pg/mL vs. 166.44 ± 38.28 pg/mL), and Ongole Grade (749.22 ± 199.30 pg/mL vs. 197.11 ± 71.20 pg/mL) (Figure 3C).

**Figure 4:**
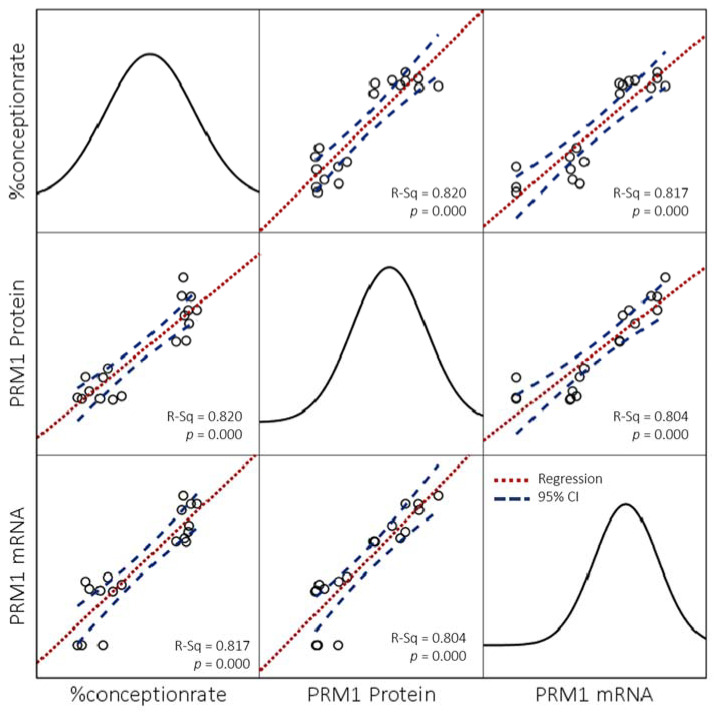
Scatter plot matrix graph of the linearity relationship between %CR and relative abundance of gene and protein of *PRM1* in bulls regardless of bull grouping based on breed and fertility.

The fertility score (%CR) in each bull breed in the study showed a positive correlation with semen quality parameters such as PM (*p* < 0.01), viability (*p* < 0.01), and plasma membrane integrity (*p* < 0.01) (Table 2). A negative correlation also existed between the %CR in all breeds of bull and the level of SDF (*p* < 0.01) (Table 2). The research results indicated that high sperm with *PRM* deficiency correlated closely with a lower %CR in all bull breeds (*p* < 0.01). Additionally, the results of the *PRM1* assessment also confirmed it at the mRNA and protein levels, with the high relative expression of genes and protein abundance indicating linearity with an increase in the %CR (Figure 4). A close correlation between %CR and *PRM1* gene expression (*p* < 0.01) and protein abundance (*p* < 0.01) was also shown in all bulls (Table 2).

## 4. Discussion

Fertile bulls are determined by the ability of a bull to produce sperm capable of fertilizing the oocytes [37] and have a new calf [38]. The fertility decline in bulls impacts cows’ low conception level, resulting in low productivity and economic problems related to the livestock industry [38,39]. Chenoweth [40] and Parkinson [41] showed that fertility data and extensive progeny records in cattle prove the inability of superior bulls to produce a maximum %CR, even when the applicable standards are met by sperm motility and morphology, similar to our study’s results. The semen used is produced from superior bulls that underwent the previous selection process with fertility scores that have not reached a good %CR category (Table 1). The quality of post-thawing semen significantly resulted in PM showing a percentage exceeding 40%, quality control of frozen semen used for the AI program in each breed of bulls in the study (Table 1). This value meets the minimum PM requirements of frozen semen used for the AI program, according to the Indonesian National Standard [3] and Zewdie et al. [42]. However, the value exceeded the standard; it did not produce a maximum fertility score. Diskin [6] and Butler [43] reported that a good fertility score reached at least 70% of 100% of the first inseminated cows.

Similar results were previously reported [4], showing that the fertility score of several Holstein bulls in Indonesia was approximately 21–35%, including in the LF bulls category. This result contradicted Rosyada et al. [4], who reported no correlation between PM and %CR (fertility); however, they showed a close correlation between the two parameters (*p* < 0.01) (Table 2). Moreover, it is undeniable that only the motile and progressive sperm can pass through the cervix and penetrate the cumulus and the zona pellucida for fertilization to occur [44].

In addition to PM, semen quality parameters such as viability and plasma membrane integrity showed a significant difference (*p* < 0.05) between HF and LF bulls (Table 1) associated with %CR (Table 2) in each breed. The outcomes of this study revealed a higher percentage value of viability and integrity of the plasma membrane than the previous research’s average [4,5]; however, it has not yet reached the maximum fertility level. This result is comparable to a previously published report by Pardede et al. [5], who discovered that even though the percentage CR was less than 70%, semen quality remained a critical aspect of bull fertility. Again, however, the results of this study aligned with the previous findings that showed the inability of conventional semen quality assessment to facilitate the fertility of a bull.

Disturbances exist during the spermatogenesis process in bulls, impacting the molecular defects that affect the quality and production of sperm, consequently decreasing fertility and even causing infertility [9]. The molecular defects in uncompensated sperm and sex-steroid enzyme alteration influence sperm’s capacity to fertilize the egg, contributing to the embryo’s normal early development [45,46,47]. Additionally, the non-compensable factors, such as molecular defects in sperm, cannot be addressed by raising the number of sperm per insemination instead of compensable characteristics, which may be solved by increasing the number of sperm per insemination [48,49,50,51]. *PRM*, the primary protein packaging for sperm DNA, is a component in sperm that plays a crucial role in determining these uncompensated traits and is also associated with molecular defects in sperm [8,10,12,13,25,28]. Additionally, hypercondensation of sperm chromatin occurs, during the elongated spermatid phase of spermiogenesis, involving the replacement of histones by transient transition proteins [52], then replaced by at least 85% by *PRM* until maternal histones return them after fertilization [53].

Proper DNA protamination of sperm influences sperm chromatin dynamics in mammals [23]. Furthermore, disturbances in this process can affect the sperm maturation process in the epididymis, causing an impact on sperm dysfunction and male subfertility, including infertility [54]. Immature sperm contains more histone-packed chromatin regions more likely to display SDF than well-*PRM* sperm [55]. Our study evaluated SDF using AO staining and the Halomax test (Figure 1). Evenson [56] reported that sperm chromatin structure assay (SCSA) is the most stable SDF assessment method, whereas AO staining is the least sensitive method compared to other methods. The Halomax test is the simplest and easiest method of evaluating SDF, which is included in a kit combined with various cell dyes primarily used with light and fluorescence microscopy [56]. The instability of sperm chromatin packaging also causes SDF and dispersion, forming a large halo [57]. Garcia-Macias et al. [34] reported that evaluating SDF using the Halomax test is one among four fertility predictors. This study did not use SCSA because of its expensiveness. It is practically not affordable for application in the field and requires flow cytometry compared to the Halomax test method and AO staining. 

The SDF assessment with AO staining showed a significant increase (*p* < 0.05) in LF bulls than in HF bulls (Figure 2A), with the threshold value reaching 3.27% in LF bulls. In a study finding, the Halomax test was used to evaluate SDF on LF bulls reporting threshold value substantially higher (*p* < 0.05) than on HF bulls (Figure 2A), with the value reaching 3.49% on LF bulls. Dogan et al. [13] reported that the Halomax test method showed a negative correlation with fertility in bulls, in low SDF bulls with LF. Our study also found that SDF percentage using both techniques was adversely linked (*p* < 0.001; *p* < 0.000) with %CR for each breed of bull (Table 2). However, previous studies reported the various effects of severe SDF, such as reduced fertility rates, poor embryo quality, and decreased pregnancy rates [58]. Cho et al. [59] and Boe-Hansen et al. [60] also reported early embryonic death and smaller fetal size due to severe SDF.

Several factors cause spermatozoa DNA fragmentation, such as hormones, age, infection, increased reactive oxygen species, chemical/toxic exposure, smoking, drugs, testicular hyperthermia, apoptosis, and protamine deficiency during spermatogenesis [51]. As previously discussed, the high SDF levels in sperm are related to the level of maturity of the sperm due to impaired protamination of the chromatin structure of sperm. However, Simon et al. [58] report showed a correlation of abnormal expression or low *PRM* content in sperm with SDF. Therefore, this study also evaluated the level of *PRM* deficiency indirectly using the CMA_3_ method (Figure 1). The guanine-cytosine-specific fluorochromes from the CMA_3_ assay competed with *PRM* binding sites on DNA, showing that chromatin packaging under *PRM* deficiency conditions will cause binding of chromomycin to DNA (CMA3+) [61]. In sperm with DNA chromatin packaging compacted and stabilized by *PRM*, arginine, which is rich in guanine-cytosine (CMA3−), abundant in *PRM*, would inhibit the dye CMA_3_ [62,63]. However, using CMA_3_ as an indirect assay of *PRM* content in sperm associated with SDF and fertility is widely used [64,65,66,67,68].

Our study’s findings agreed with earlier research, where LF bulls in each bull breed exhibited a higher percentage of CMA3+ or *PRM* deficiency (*p* < 0.01) than HF bulls (Figure 3A). A negative correlation (*p* < 0.000) shown between CMA3+ and the %CR in all breeds of bulls was used in this study (Table 2). In this study, the results of the indirect *PRM* assessment were also confirmed by identifying *PRM1*, both at the mRNA (Figure 3B) and protein levels (Figure 3C). *PRM1* mRNA was highly expressed (*p* < 0.05) in HF bulls than in LF bulls in all bull breeds during this study (Figure 3B). At the protein level, *PRM1* protein was also seen to be significantly more abundant (*p* < 0.05) in HF bulls compared to LF bulls in all breeds of bulls (Figure 3C). A positive correlation (*p* < 0.000; *p* < 0.009) (Table 2) and linearity in the linear regression curve graph (Figure 4) were also shown between mRNA expression and *PRM1* protein abundance with %CR. Our results are similar to those reported in a previous study in Holstein bulls, showing transcription of *PRM1* mRNA with low concentrations in LF bulls [12]. Additionally, Dogan et al. [13] reported that *PRM1* was detected in low concentrations in bulls with LF rates with an increased SDF at the protein level.

The condition of *PRM* deficiency or aberrant protamination of sperm resulting in LF conditions or even infertility cases is associated with several plausible causes, including disruption of transcription, translation, and synthesis processes [69,70,71,72]. Recently, research has linked defective protein synthesis to abnormal mRNA retention, implying that abnormalities in *PRM* translation control may lead to *PRM* inadequacy in infertile males [73]. Additionally, it is reported that abnormal histone ratios resulting from the failure or complete failure of the protein remodeling process in sperm chromatin, in which *PRM* dominates 85% of the sperm nucleus protein, is a cause of *PRM* aberration that causes male infertility [74]. In other species, *PRM* haploinsufficiency in transgenic mice reportedly impacts changes or disruption of spermatogenesis and result in infertility [75]. However, unlike in mice and humans, where another variant of *PRM*, PRM2, must have a similar ratio as *PRM1*, and a ratio that deviates at the protein and mRNA levels, will impact infertility [76,77,78,79]. *PRM1* in bulls is the only *PRM* variant that plays a direct role in the normal function of fertile sperm [12,13]. Ganguly et al. [27] and Pardede et al. [20] also proved that *PRM1*, both at the mRNA and protein levels, was the most critical *PRM* variant in normal sperm function in bulls.

Nevertheless, specifically, the regulation and mechanism of *PRM1* in sperm remains not fully understood [29] necessary to be studied further. Steger et al. [80] stated that abnormal expression or decreased expression and concentration of *PRM1* resulting from the abnormal function of transcriptional regulators, translation, or post-translational modifications affect *PRM* and various genes during spermatogenesis. Therefore, it is not surprising that *PRM* can be a “checkpoint” during spermiogenesis, and abnormal *PRM* expression will impact the rate of apoptosis [80]. Increased apoptosis affects poor semen quality associated with fertilization and pregnancy after IVF [81]. However, this study supports the hypotheses that have been reported so far regarding the importance of *PRM1* in normal spermatozoa function and its relationship to fertility in bulls, especially bulls used for AI programs in Indonesia. As found in this study, *PRM1* proved to be closely related to fertility, with bulls still classified as subfertile or LF, even though the semen quality was conventionally categorized as suitable for the AI program. Moreover, the combination of assessment with a gene or protein analysis approach with semen quality assessment, including SDF assessment, can be the best choice in determining the fertility of bulls.

## 5. Conclusions

In conclusion, inadequate or low sperm chromatin protamination at both mRNA and protein levels was associated with defects in sperm chromatin condensation, found with increased SDF, and coinciding with a decrease in the %CR (fertility score) in bulls. Therefore, considering that this research aimed to find candidate fertility markers that accurately determine bulls’ fertility and can be used in the bull selection process, especially in Indonesia, *PRM1* shows to be a promising candidate in terms of fertility markers. Furthermore, to the best of our knowledge, this is the first work in Indonesia to demonstrate correlations in the mRNA expression and protein content of sperm *PRM1* from HF compared to LF bulls. Moreover, it is not only in one breed of cattle but also represents commodity groups of cattle breeds in Indonesia, such as exotic beef cattle (Limousin), dairy cattle (Holstein), and local cattle breeds (Ongole Grade).

## Figures and Tables

**Table 1:** The difference in the Limousin, Holstein, and Ongole Grade bulls mean parameters, with different fertility, HF vs. LF bulls.

Parameter	Breed	HF Bulls Mean (±SD)		LF Bulls Mean (±SD)	
Fertility score (%CR)	Limousin	80.13	(±0.82) *	55.44	(±3.53)
	Holstein	80.87	(±1.44) *	54.75	(±2.88)
	Ongole Grade	78.17	(±0.97) *	60.71	(±2.42)
Progressive Motility (%)	Limousin	56.01	(±3.39) *	48.56	(±2.29)
	Holstein	53.26	(±2.77) *	45.40	(±1.50)
	Ongole Grade	49.85	(±3.01) *	44.29	(±1.97)
Sperm Viability (%)	Limousin	78.73	(±3.32) *	71.67	(±1.53)
	Holstein	80.30	(±2.99) *	72.16	(±3.71)
	Ongole Grade	74.80	(±3.68) *	68.47	(±2.64)
Plasma Membran Integrity (%)	Limousin	79.10	(±3.55) *	71.87	(±1.23)
	Holstein	79.70	(±1.79) *	72.93	(±3.21)
	Ongole Grade	74.80	(±3.47) *	68.57	(±2.41)

* Significant difference when compared to LF (*p* < 0.05).

**Table 2:** Correlation of bull fertility scores with sperm quality parameters, SDF, *PRM1* gene, and protein abundance in Limousin, Holstein, and Ongole Grade bulls ^a^.

Breed	Parameter	Correlation Coefficient	*p*-Value
Limousin	%CR vs. PM (%)	0.786	<0.000
	%CR vs. viability (%)	0.821	<0.000
	%CR vs. PMI (%)	0.810	<0.000
	%CR vs. AO (%)	−0.776	<0.000
	%CR vs. Halomax test (%)	−0.694	<0.001
	%CR vs. CMA3+ (%)	−0.818	<0.000
	%CR vs. *PRM1* gene	0.894	<0.000
	%CR vs. *PRM1* protein (pg/mL)	0.595	<0.009
Holstein	%CR vs. PM (%)	0.872	<0.000
	%CR vs. viability (%)	0.784	<0.000
	%CR vs. PMI (%)	0.809	<0.000
	%CR vs. AO (%)	−0.725	<0.000
	%CR vs. Halomax test (%)	−0.671	<0.002
	%CR vs. CMA3+ (%)	−0.753	<0.000
	%CR vs. *PRM1* gene	0.939	<0.000
	%CR vs. *PRM1* protein (pg/mL)	0.737	<0.000
Ongole Grade	%CR vs. PM (%)	0.707	<0.000
	%CR vs. viability (%)	0.681	<0.000
	%CR vs. PMI (%)	0.704	<0.000
	%CR vs. AO (%)	−0.834	<0.000
	%CR vs. Halomax test (%)	−0.769	<0.000
	%CR vs. CMA3+ (%)	−0.826	<0.000
	%CR vs. *PRM1* gene	0.931	<0.000
	%CR vs. *PRM1* protein (pg/mL)	0.884	<0.000

^a^ The Pearson correlation coefficient (r) and *p*-value (*p* < 0.01) represent all values obtained, regardless of the grouping of bulls based on fertility. %CR: conception rate; PM: progressive motility; PMI: plasma membrane integrity; AO: acridine orange.

## Data Availability

The data that support the findings of this study are available from the corresponding author, B.P.P., upon reasonable request.

## References

[1] Verberckmoes S., Van Soom A., de Kruif A. (2004). Intra-uterine insemination in farm animals and humans. Reprod. Domest. Anim..

[2] Pardede B.P., Supriatna I., Yudi Y., Agil M. (2020). Decreased bull fertility: Age-related changes in sperm motility and DNA fragmentation. E3S Web Conf..

[3] BSN (2017). National Standardization of Bull Frozen Semen Part 1. SNI No. 4869.1.

[4] Rosyada Z.N.A., Ulum M.F., Tumbelaka L.I.T.A., Purwantara B. (2020). Sperm protein markers for Holstein bull fertility at National Artificial Insemination Centers in Indonesia. Vet. World.

[5] Pardede B.P., Agil M., Yudi Y., Supriatna I. (2020). Relationship of frozen-thawed semen quality with the fertility rate after being distributed in the Brahman Cross Breeding Program. Vet. World.

[6] Diskin M.G. (2014). Achieving High Reproductive Performance in Beef Herds.

[7] Mishra C., Palai T.K., Sarangi L.N., Prusty B.R., Maharana B.R. (2013). Candidate gene markers for sperm quality and fertility in bulls. Vet. World.

[8] Özbek M., Hitit M., Kaya A., Jousan F.D., Memili E. (2021). Sperm functional genome associated with bull fertility. Front. Vet. Sci..

[9] Staub C., Johnson L. (2018). Review: Spermatogenesis in the bull. Animal.

[10] Pardede B.P., Agil M., Supriatna I. (2020). Protamine and other proteins in sperm and seminal plasma as molecular markers of bull fertility. Vet. World.

[11] Moura A.A., Memili E. (2016). Functional aspects of seminal plasma and sperm proteins and their potential as molecular markers of fertility. Anim. Reprod..

[12] Feugang J.M., Rodriguez-Osorio N., Kaya A., Wang H., Page G., Ostermeier G.C., Topper E.K., Memili E. (2010). Transcriptome analysis of bull spermatozoa:Implications for male fertility. Reprod. Biomed. Online.

[13] Dogan S., Vargovic P., Oliveira R., Belser L.E., Kaya A., Moura A., Sutovsky P., Parrish J., Topper E., Memili E. (2015). Sperm protamine-status correlates to the fertility of breeding bulls. Biol. Reprod..

[14] Ward W.S. (2010). Function of sperm chromatin structural elements in fertilization and development. Mol. Hum. Reprod..

[15] Bao J., Bedford M.T. (2016). Epigenetic regulation of the histone-to-protamine transition during spermiogenesis. Reproduction.

[16] Wang T., Gao H., Li W., Liu C. (2019). Essential role of histone replacement and modifications in male fertility. Front. Genet..

[17] O’Donnell L. (2015). Mechanisms of spermiogenesis and spermiation and how they are disturbed. Spermatogenesis.

[18] Bower P.A., Yelick P.C., Hecht N.B. (1987). Both P1 and P2 protamine genes are expressed in mouse, hamster, and rat. Biol. Reprod..

[19] Corzett M., Mazrimas J., Balhorn R. (2002). Protamine 1: Protamine 2 stoichiometry in the sperm of eutherian mammals. Mol. Reprod. Dev..

[20] Pardede B.P., Maulana T., Kaiin E.M., Agil M., Karja N.W.K., Sumantri C., Supriatna I. (2021). The potential of sperm bovine protamine as a protein marker of semen production and quality at the National Artificial Insemination Center of Indonesia. Vet. World.

[21] Maier W.M., Nussbaum G., Domenjoud L., Klemm U., Engel W. (1990). The lack of protamine 2 (P2) in boar and bull spermatozoa is due to mutations within the P2 gene. Nucleic Acids Res..

[22] Ferraz M.A.M., Simoes R., Barros F.O., Millazzoto M.P., Visintin J.A., Assumpcao M.E.O.D. (2013). Gene expression profle of protamines and transition nuclear proteins in bovine testis. Braz. J. Vet. Res. Anim. Sci..

[23] Champroux A., Torres-Carreira J., Gharagozloo P., Drevet J.R., Kocer A. (2016). Mammalian sperm nuclear organization: Resiliencies and vulnerabilities. Basic Clin. Androl..

[24] Champroux A., Cocquet J., Henry-Berger J., Drevet J.R., Kocer A. (2018). A decade of exploring the mammalian sperm epigenome: Paternal epigenetic and transgenerational inheritance. Front. Cell. Dev. Biol..

[25] Fortes M.R., Satake N., Corbet D.H., Corbet N.J., Burns B.M., Moore S.S., Boe-Hansen G.B. (2014). Sperm protamine deficiency correlates with sperm DNA damage in Bos indicus bulls. Andrology.

[26] Francism S., Yelumalai S., Jones C., Coward K. (2014). Aberrant protamine content in sperm and consequential implications for infertility treatment. Hum. Fertil..

[27] Ganguly I., Gaur G.K., Kumar S., Mandal D.K., Kumar M., Singh U., Kumar S., Sharma A. (2013). Differential expression of protamine 1 and 2 genes in mature spermatozoa of normal and motility impaired semen producing crossbred Frieswal (HF×Sahiwal) bulls. Res. Vet. Sci..

[28] Aoki V.W., Liu L., Jones K.P., Hatasaka H.H., Gibson M., Peterson C.M., Carrell D.T. (2006). Sperm protamine 1/protamine 2 ratios are related to in vitro fertilization pregnancy rates and predictive of fertilization ability. Fertil. Steril..

[29] Takeda N., Yoshinaga K., Furushima K., Takamune K., Li Z., Abe S., Aizawa S., Yamamura K. (2016). Viable offspring obtained from Prm1-deficient sperm in mice. Sci. Rep..

[30] Nemati H., Sadeghi M., Nazeri M., Mohammadi M. (2020). Evaluation of the association between polymorphisms of *PRM1* and PRM2 and the risk of male infertility: A systematic review, meta-analysis, and meta-regression. Sci. Rep..

[31] Rodríguez-Martínez H., Chenoweth P.J. (2000). Evaluation of frozen semen: Traditional and new approaches. Topics in Bull Fertility.

[32] Jeyendran R.S., Van der Ven H.H., Perez-Pelaez M., Crabo B.G., Zaneveld L.J. (1984). Development of an assay to assess the functional integrity of the human sperm membrane and its relationship to other semen characteristics. J. Reprod. Fertil..

[33] Mohammed E.E., Mosad E., Zahran A.M., Hameed D.A., Taha E.A., Mohamed M.A. (2015). Acridine orange and flow cytometry: Which is better to measure the effect of varicocele on sperm DNA integrity?. Adv. Urol..

[34] García-Macías V., de Paz P., Martinez-Pastor F., Alvarez M., Gomes-Alves S., Bernardo J., Anel E., Anel L. (2007). DNA fragmentation assessment by flow cytometry and Sperm-Bos-Halomax (bright-field microscopy and fluorescence microscopy) in bull sperm. Int. J. Androl..

[35] Abdillah D.A., Setyawan E.M.N., Oh H.J., Ra K., Lee S.H., Kim M.J., Lee B.C. (2019). Iodixanol supplementation during sperm cryopreservation improves protamine level and reduces reactive oxygen species of canine sperm. J. Vet. Sci..

[36] Schmittgen T.D., Livak K.J. (2008). Analyzing real-time PCR data by the comparative C(T) method. Nat. Protoc..

[37] Allouche L., Madani T., Mechmeche M., Clement L., Bouchemal A. (2017). Bull fertility and its relation with density gradient selected sperm. Int. J. Fertil. Steril..

[38] Butler M.L., Bormann J.M., Weaber R.L., Grieger D.M., Rolf M.M. (2019). Selection for bull fertility: A review. Transl. Anim. Sci..

[39] Walsh S.W., Williams E.J., Evans A.C. (2011). A review of the causes of poor fertility in high milk producing dairy cows. Anim. Reprod. Sci..

[40] Chenoweth P.J. (2007). Influence of the male on embryo quality. Theriogenology.

[41] Parkinson T.J. (2004). Evaluation of fertility and infertility in natural service bulls. Vet. J..

[42] Zewdie E., Deneke N., Fikre-Mariam D., Chaka E., Haile-Mariam D., Mussa A. (2005). Guidelines and Procedures on Bovine Semen Production.

[43] Butler S. (2014). Dairy Cow Reproduction.

[44] Henkel R. (2012). Sperm preparation: State-of-the-art--physiological aspects and application of advanced sperm preparation methods. Asian J. Androl..

[45] De Jonge C. (1999). Attributes of fertile spermatozoa: An update. J. Androl..

[46] Rosati L., Di Fiore M.M., Prisco M., Di Giacomo Russo F., Venditti M., Andreuccetti P., Chieffi Baccari G., Santillo A. (2019). Seasonal expression and cellular distribution of star and steroidogenic enzymes in quail testis. J. Exp. Zool. B Mol. Dev. Evol..

[47] Di Lorenzo M., Mileo A., Laforgia V., De Falco M., Rosati L. (2021). Alkyphenol exposure alters steroidogenesis in male lizard *Podarcis siculus*. Animals.

[48] Chenoweth P.J. (2005). Genetic sperm defects. Theriogenology.

[49] Okabe M., Ikawa M., Ashkenas J. (1998). Male infertility and the genetics of spermatogenesis. Am. J. Hum. Genet..

[50] Zheng H., Stratton C.J., Morozumi K., Jin J., Yanagimachi R., Yan W. (2007). Lack of Spem1 causes aberrant cytoplasm removal, sperm deformation, and male infertility. Proc. Natl. Acad. Sci. USA.

[51] Zini A., Libman J. (2006). Sperm DNA damage: Importance in the era of assisted reproduction. Curr. Opin. Urol..

[52] Chapman J.C., Michael S.D. (2003). Proposed mechanism for sperm chromatin condensation/decondensation in the male rat. Reprod. Biol. Endocrinol..

[53] Carrell D.T., Liu L. (2001). Altered protamine 2 expression is uncommon in donors of known fertility, but common among men with poor fertilizing capacity, and may reflect other abnormalities of spermiogenesis. J. Androl..

[54] Shamsi M.B., Kumar K., Dada R. (2011). Genetic and epigenetic factors: Role in male infertility. Indian J. Urol..

[55] Schulte R.T., Ohl D.A., Sigman M., Smith G.D. (2010). Sperm DNA damage in male infertility: Etiologies, assays, and outcomes. J. Assist. Reprod. Genet..

[56] Evenson D.P. (2016). The Sperm Chromatin Structure Assay (SCSA(^®^)) and other sperm DNA fragmentation tests for evaluation of sperm nuclear DNA integrity as related to fertility. Anim. Reprod. Sci..

[57] Pourmasumi S., Nazari A., Fagheirelahee N., Sabeti P. (2019). Cytochemical tests to investigate sperm DNA damage: Assessment and review. J. Fam. Med. Prim. Care.

[58] Simon L., Castillo J., Oliva R., Lewis S.E. (2011). Relationships between human sperm protamines, DNA damage and assisted reproduction outcomes. Reprod. Biomed. Online.

[59] Cho C., Jung-Ha H., Willis W.D., Goulding E.H., Stein P., Xu Z., Schultz R.M., Hecht N.B., Eddy E.M. (2003). Protamine 2 deficiency leads to sperm DNA damage and embryo death in mice. Biol. Reprod..

[60] Boe-Hansen G.B., Christensen P., Vibjerg D., Nielsen M.B., Hedeboe A.M. (2008). Sperm chromatin structure integrity in liquid stored boar semen and its relationships with field fertility. Theriogenology.

[61] Bianchi P.G., Manicardi G.C., Urner F., Campana A., Sakkas D. (1996). Chromatin packaging and morphology in ejaculated human spermatozoa: Evidence of hidden anomalies in normal spermatozoa. Mol. Hum. Reprod..

[62] Tavalaee M., Kiani A., Arbabian M., Deemeh M.R., Esfahani M.H.N. (2010). Flow cytometry: A new approach for indirect assessment of sperm protamine deficiency. Int. J. Fertil. Steril..

[63] Fathi Z., Tavalaee M., Kiani A., Deemeh M.R., Modaresi M., Nasr-Esfahani M.H. (2011). Flow Cytometry: A Novel Approach for Indirect Assessment of Protamine Deficiency by CMA3 Staining, Taking into Account the Presence of M540 or Apoptotic Bodies. Int. J. Fertil. Steril..

[64] Bianchi P.G., Manicardi G.C., Bizzaro D., Bianchi U., Sakkas D. (1993). Effect of deoxyribonucleic acid protamination on fluorochrome staining and in situ nick-translation of murine and human mature spermatozoa. Biol. Reprod..

[65] Esterhuizen A.D., Franken D.R., Lourens J.G., Prinsloo E., van Rooyen L.H. (2000). Sperm chromatin packaging as an indication of in-vitro fertilization rates. Hum. Reprod..

[66] Nasr-Esfahani M.H., Razavi S., Mozdarani H., Mardani M., Azvagi H. (2004). Relationship between protamine deficiency with fertilization rate and incidence of sperm premature chromosomal condensation post-ICSI. Andrologia.

[67] Zandemami M., Qujeq D., Akhondi M.M., Kamali K., Raygani M., Lakpour N., Shiraz E.S., Sadeghi M.R. (2012). Correlation of CMA3 staining with sperm quality and protamine deficiency. Science.

[68] Carreira J.T., Trevizan J.T., Carvalho I.R., Kipper B., Rodrigues L.H., Silva C., Perri S.H.V., Drevet J.R., Koivisto M.B. (2017). Does sperm quality and DNA integrity differ in cryopreserved semen samples from young, adult, and aged Nellore bulls?. Basic Clin. Androl..

[69] Olivia R. (2006). Protamines and male infertility. Hum. Reprod. Update.

[70] de Oliveira R.V., Dogan S., Belser L.E., Kaya A., Topper E., Moura A., Thibaudeau G., Memili E. (2013). Molecular morphology and function of bull spermatozoa linked to histones and associated with fertility. Reproduction.

[71] Carrell D.T., Emery B.R., Hammoud S. (2007). Altered protamine expression and diminished spermatogenesis: What is the link?. Hum. Reprod. Update.

[72] Aoki V.W., Carrell D.T. (2003). Human protamines and the developing spermatid: Their structure, function, expression and relationship with male infertility. Asian J. Androl..

[73] Aoki V.W., Liu L., Carrell D.T. (2006). A novel mechanism of protamine expression deregulation highlighted by abnormal protamine transcript retention in infertile human males with sperm protamine deficiency. Mol. Hum. Reprod..

[74] Zhang X., San Gabriel M., Zini A. (2006). Sperm nuclear histone to protamine ratio in fertile and infertile men: Evidence of heterogeneous subpopulations of spermatozoa in the ejaculate. J. Androl..

[75] Cho C., Willis W.D., Goulding E.H., Jung-Ha H., Choi Y.C., Hecht N.B., Eddy E.M. (2001). Haploinsufficiency of protamine-1 or -2 causes infertility in mice. Nat. Genet..

[76] Steger K., Failing K., Klonisch T., Behre H.M., Manning M., Weidner W., Hertle L., Bergmann M., Kliesch S. (2001). Round spermatids from infertile men exhibit decreased protamine-1 and -2 mRNA. Hum. Reprod..

[77] Steger K., Fink L., Failing K., Bohle R.M., Kliesch S., Weidner W., Bergmann M. (2003). Decreased protamine-1 transcript levels in testes from infertile men. Mol. Hum. Reprod..

[78] Balhorn R., Reed S., Tanphaichitr N. (1988). Aberrant protamine 1/protamine 2 ratios in sperm of infertile human males. Experientia.

[79] Belokopytova I.A., Kostyleva E.I., Tomilin A.N., Vorob’ev V.I. (1993). Human male infertility may be due to a decrease of the protamine P2 content in sperm chromatin. Mol. Reprod. Dev..

[80] Steger K., Wilhelm J., Konrad L., Stalf T., Greb R., Diemer T., Kliesch S., Bergmann M., Weidner W. (2008). Both protamine-1 to protamine-2 mRNA ratio and Bcl2 mRNA content in testicular spermatids and ejaculated spermatozoa discriminate between fertile and infertile men. Hum. Reprod..

[81] Oosterhuis G.J., Vermes I. (2004). Apoptosis in human ejaculated spermatozoa. J. Biol. Regul. Homeost. Agents.

